# Analytical Strategy to Prioritize Alzheimer’s Disease Candidate Genes in Gene Regulatory Networks Using Public Expression Data

**DOI:** 10.3233/JAD-170011

**Published:** 2017-08-14

**Authors:** Shweta Bagewadi Kawalia, Tamara Raschka, Mufassra Naz, Ricardo de Matos Simoes, Philipp Senger, Martin Hofmann-Apitius

**Affiliations:** a Fraunhofer Institute for Algorithms and Scientific Computing (SCAI), Schloss Birlinghoven, Sankt Augustin, Germany; b Rheinische Friedrich-Wilhelms-Universität Bonn, Bonn-Aachen International Center for Information Technology, Bonn, Germany; c University of Applied Sciences Koblenz, RheinAhrCampus, Remagen, Germany; d Dana-Farber Cancer Institute, Boston, MA, USA

**Keywords:** Alzheimer’s disease, gene regulatory networks, microarray analysis, synaptic transmission

## Abstract

Alzheimer’s disease (AD) progressively destroys cognitive abilities in the aging population with tremendous effects on memory. Despite recent progress in understanding the underlying mechanisms, high drug attrition rates have put a question mark behind our knowledge about its etiology. Re-evaluation of past studies could help us to elucidate molecular-level details of this disease. Several methods to infer such networks exist, but most of them do not elaborate on context specificity and completeness of the generated networks, missing out on lesser-known candidates. In this study, we present a novel strategy that corroborates common mechanistic patterns across large scale AD gene expression studies and further prioritizes potential biomarker candidates. To infer gene regulatory networks (GRNs), we applied an optimized version of the BC3Net algorithm, named BC3Net10, capable of deriving robust and coherent patterns. In principle, this approach initially leverages the power of literature knowledge to extract AD specific genes for generating viable networks. Our findings suggest that AD GRNs show significant enrichment for key signaling mechanisms involved in neurotransmission. Among the prioritized genes, well-known AD genes were prominent in synaptic transmission, implicated in cognitive deficits. Moreover, less intensive studied AD candidates (STX2, HLA-F, HLA-C, RAB11FIP4, ARAP3, AP2A2, ATP2B4, ITPR2, and ATP2A3) are also involved in neurotransmission, providing new insights into the underlying mechanism. To our knowledge, this is the first study to generate knowledge-instructed GRNs that demonstrates an effective way of combining literature-based knowledge and data-driven analysis to identify lesser known candidates embedded in stable and robust functional patterns across disparate datasets.

## INTRODUCTION

Alzheimer’s disease (AD) is a very complex idiopathic disease contributing to immense personal and societal burden, with ∼13.8 million people being affected by 2050 [1] in the US alone. High failure rate of AD drugs (98%) in Phase III trials have resulted in no new FDA approved drugs since 2003 [2]. Moreover, the five previously approved AD drugs just provide symptomatic relief [3]. Not all, but a substantial proportion of these studies focused on amyloid-β (Aβ) and tau accumulations as being synonymous to the AD pathology [4], leading to an unprecedented wealth of molecular and clinical data. Despite the disappointing outcome of the clinical trials, neurology researchers still believe in the definiteness of these two hypotheses [5]. This reaffirms that pathological mechanisms underlying AD are much more complex than the current consideration, thus, opening up possibilities for new therapeutic targets. Working toward unraveling dysregulated events heralding known and unknown patterns could fill the gaps between AD hallmarks [6].

Existing experimental data, not being fully exploited, contain compelling evidence that have the potential to contribute next groundbreaking discoveries. The great challenge, however, lies in harmoniously integrating these data and interpreting them differently to derive new-novel insights while maintaining the biological connections. The term “Horizontal Meta-analysis” implies the integration of results from several independent studies [7], thereby increasing the statistical power of the derived conclusion. A more conventional gene-centric approach is to intercross differentially expressed (DE) genes across studies based on majority voting [8], merging gene ranks [9], and combining *p*-values [8]. However, differing factors can lead to a low overlap and discrepancies between studies such as the applied statistical methods, different platforms of the quantitative measurements, and heterogeneity of the patient cohorts [10]. Moreover, these approaches do not shed light on the coordinated genes that collectively orchestrates the underlying (patho-)mechanism. A more consistent and robust approach is through functional enrichment of the dysregulated genes using KEGG [11], MSigDB [12], and other sources of pathway knowledge. However, these approaches have a tendency to converge toward genes that express in large magnitudes and generated hypotheses are restricted by current understanding of pathways. Network-based approaches that rely on the coherence of expression changes between functionally dependent genes could provide an effective means to overcome the above-mentioned challenges. Such inferred networks have the capability to determine subtle expression shifts between correlated gene pairs that are linked to the dysregulation events. Particularly, these signatures are largely consistent across different studies; thus, emphasizing on its benefits for large scale meta-analysis. In the last few years, we have seen a swarm of methods that infer such networks based on co-expression, regulation, and causal information namely WGCNA [13], BC3Net [14], MRNET [15], ARACNE [16], GENIE3 [17], and CLR [18]. Among these, WGCNA and the bagging version of C3Net (BC3Net) are popular and computationally efficient methods. BC3Net is an ensemble method that statistically infers GRNs based on the strongest mutual dependencies between genes, whereas WGCNA clusters genes on the basis of calculated pairwise correlation coefficients. In the meantime, BC3Net has been reported in providing meaningful biological insights for large-scale studies [19, 20].

Traditional GRNs identify patterns through differential co-expression analysis [21–23], displaying grouping of patterns based on dysregulated and coordinated biomolecular changes. Integration of such priors drastically improves the context specificity of the inferred networks relative to using data as the sole source [24–26]. However, these spurious discriminative structures, in a given disease context, may vary since DE genes are highly inconsistent across studies [27]. Biologically speaking, one may argue that the differences in functionally enriched components, derived from DE genes, are more consistent than gene-centric activities [28]. But this approach misses out on less informative and less studied non-DE genes, which act in groups, contributing to the observed phenotype or a part of cascade effect. Furthermore, overlaying the inferred networks with known interactions, cataloged in databases or harvested from published literature expand the knowledge space [29, 30]. However, an intriguing question on completeness, veracity and context specificity of these interactions has proven to be a major setback [31].

**Fig.1:**
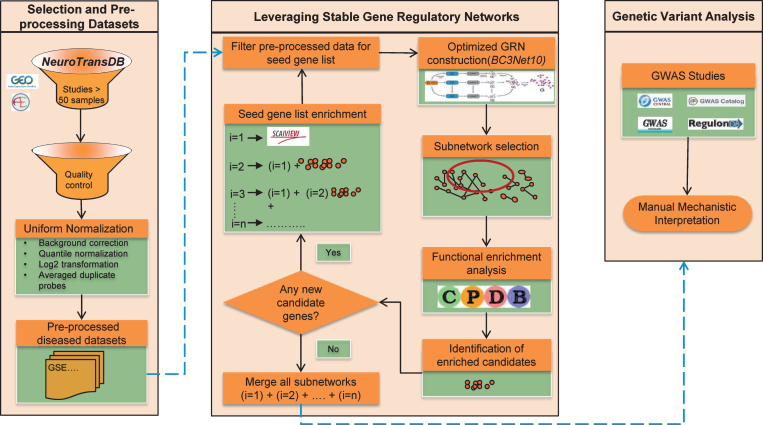
The overall strategy applied to obtain robust gene expression patterns across public Alzheimer’s disease studies. Firstly, four gene expression datasets were shortlisted from NeuroTransDB database. The selected studies underwent preprocessing and quality control. In each dataset, the intensity values were limited to the seed gene list. To enrich the seed, functional enrichment was applied where genes from the identified significant pathways from each dataset’s subnetwork (edge weight >0.5), generated using BC3Net10 approach, were included. When no additional genes were identified, subnetworks from each iteration, separately for each dataset, were merged into an aggregated network for further prioritization of the genes using genetic variant analysis.

Here we propose a new approach to identify common signature patterns across public AD studies and prioritize lesser known AD candidates that unravel the general principles of the intrinsic patho-mechanisms. To identify AD mechanistic footprints, we established an optimized workflow around BC3Net to extract more robust and coherent co-expressed gene patterns (named BC3Net10). The approach allows us to converge lesser known candidates into the final generated GRNs. Moreover, to generate context-specific GRNs, the main rationale applied was to leverage the power of prior knowledge and functionally enriched candidates in the data. First, we identified the most frequently discussed genes in the scientific literature using our literature mining environment SCAIView; this is called the “seed”. We are aware that the generated GRNs may be biased due to the incomplete nature of the prior knowledge. To overcome this limitation, we extended the seed by adding all the genes from the enriched pathways, determined for the high scoring inferred interactions from BC3Net10. Several iterations are performed until there are no more genes to be added to the seed. Finally, an aggregated GRN from all the iterations, for each dataset, is generated to prioritize functional context and determine lesser known candidates from the genetic variant analysis. Figure 1 presents an overview of the strategy in this study, and descriptions of the methodology are available in the Material and Methods section. This work suggests a context-specific strategy for future interpretation of the GRNs. Taken together, our work demonstrates that optimizing the GRN generation can provide a powerful resource to prioritize novel candidate genes (could serve as biomarkers) and common functional components that axles the disease progression.

## MATERIAL AND METHODS

### Selection of datasets

We collated eight AD datasets (cf. Table 1) that are composed of 50 or more samples (for diseased and control phenotype) from the previously developed value-added database, *NeuroTransDB*. Briefly, this database contains manually curated metadata annotations for eligible neurodegenerative studies. The datasets have been harvested from publicly available resources namely, Gene Expression Omnibus (GEO) [33] and ArrayExpress [34], using a keyword-based search approach.

**Table 1:** List of datasets shortlisted from NeuroTransDB database for generating gene regulatory networks. Final selected studies are highlighted in bold

GEO ID	Number of Samples		Sample Source	Stage	Platform
	Diseased	Control
**GSE5281**	87	74	Entorhinal cortex, Hippocampus,	–	Affymetrix HG U133 Plus 2
			Primary visual cortex, Prefrontal
			cortex, Medial temporal gyrus,
			Superior frontal gyrus
**GSE44768**	129	101	Cerebellum	LOAD	Rosetta/Merck Human 44k 1.1 microarray
**GSE44771**	129	101	Visual cortex	LOAD	Rosetta/Merck Human 44k 1.1 microarray
**GSE44770**	129	101	Dorsolateral prefrontal cortex	LOAD	Rosetta/Merck Human 44k 1.1 microarray
GSE13214	52	40	Hippocampal, Cortex frontal	Braak 4–6	Homo sapiens 4.8K 02-01 amplified cDNA
GSE15222	176	187	Cortical tissue	LOAD	Sentrix HumanRef-8 Expression BeadChip
GSE29676	350	200	Blood	–	Invitrogen ProtoArray v5.0
GSE33528	615	600	Blood	LOAD	Illumina Human- Hap650Yv2 Genotyping BeadChip

Furthermore, datasets that fulfilled the following criteria were retained for generating gene regulatory networks: (1) oligonucleotide arrays for analysis consistency, (2) availability of raw data to facilitate uniform pre-processing, and (3) expression profiling carried out on brain tissue. A list of four potential datasets that comply with the above conditions is eligible for further analysis: GSE5281, GSE44771, GSE44770, and, GSE44768. An overview of the platform, stage, and brain region information for the same is given in Table 1. Among these, GSE44771, GSE44770, and GSE44768 were from a single study reported by Zhang et al. [35] for late-onset AD.

### Pre-processing and gene annotation

The four selected datasets were processed identically to reduce variance and to maintain consistent quality. All analysis was carried out with R (Version 3.1.3) [36], an open-source statistical language, using the packages from Bioconductor (Version 3.0) [37]. The overall step-by-step workflow is shown in Fig. 1. To eliminate the variance effect of non-specific hybridization, all the downloaded raw data were uniformly normalized by performing background correction, quantile normalization, and averaging the expression values of duplicate probes on log2-transformed intensity values. For Affymetrix platform, robust multi-array average method (*rma*) [38] available in Bioconductor package *affy* was applied. Similar methods available in Bioconductor package *limma* [39] were applied on Rosetta/Merck Human 44k 1.1 microarray chip.

Affymetrix probes to gene symbols annotation mapping were obtained from the “hgu133plus2.db” Bioconductor package. In the case of Rosetta/Merck chip, the gene symbol annotations were provided directly along with the intensity values. For multiple probes mapping to the same gene within an array, average expression values were used. Unmapped probes were excluded from further analyses. As a result of this preprocessing step, we retained 20155 in GSE5281, 11254 in GSE44771, 10437 in GSE44770, and 12000 in GSE44768 genes for further analysis.

### Quality control and outlier detection

Using the Bioconductor package *arrayQualityMetrics* [40], we assessed the array quality and removed the outlier samples. Describing shortly, *arrayQualityMetrics* determine outliers using three different metrics: (1) distance between samples using principal component analysis, (2) array intensity distributions of all samples on the array; and (3) individual array quality through MA-plots. If a sample is detected as an outlier in either of the three metrics, we discard it from further analysis. In the four selected datasets, 9 in GSE5281, 19 in GSE44771, 27 in GSE44770, and 12 in GSE44768 arrays were outliers. The list of identified outlier arrays is provided in the Supplementary File 3. The remaining arrays that passed the quality control were processed as described earlier.

### Leveraging stable gene regulatory networks

In order to derive AD relevant GRNs, we divided the AD gene expression profile based on their phenotypes, disease and normal. Subsequently, BC3Net10 algorithm was applied only on diseased samples for AD seed genes, cf. Fig. 1. GRNs were generated independently for each dataset, visualized as igraph objects in Cytoscape tool [41]. Network topological properties such as node degree, hub genes, etc. were determined using the Bioconductor package igraph.

#### Filter pre-processed data for seed gene list

Prior to GRN generation, each pre-processed dataset was restricted to the genes in the seed. Initially, it consists of a set of literature-derived genes that have high probability of direct or indirect involvement in AD pathogenesis (see the section on Gathering initial seed genes). The rationale behind applying this filtration is to maintain the disease specificity and reduce high run time due to bootstrapping in BC3NET. Further on, after every functional enrichment iteration, we again restrict the expression data to the new seed.

#### Gathering initial seed genes

The backbone of the seed comprises of the results harnessed from our text-mining knowledge framework, SCAIView [42]. SCAIView is a knowledge discovery framework that supports named entity recognition, information retrieval, and information extraction on large textual sources. Its capability to rank documents and biomedical entities based on the relevancy score allows retrieval of significant players in a disease context [43, 44]. Querying SCAIView for AD related genes resulted in 4808 genes, as of 2 January 2016. Only the top 500 retrieved genes were used as the initial seed, depicted as i = 1 in Fig. 1.

#### Optimized GRN construction

For the construction of GRNs, R package *bc3net* was applied to the processed data with 100 bootstraps (B = 100). Briefly explained, one aggregated network was generated by applying the C3Net algorithm on 100 bootstrapped data, which were inferred from given processed dataset. Statistically, non-significant edges inferred by C3Net and BC3Net were discarded using Bonferroni’s multiple testing correction, α= 0.05. In the resulting aggregated network, edge weights represent the frequency of a correlated gene pair in 100 random sampling, ranging from 0 to 1.

During random sampling, true and most prominent correlations are stochastically more likely to be selected than the non-correlated ones. This is reflected in BC3Net networks, where three independently generated GRNs, inferred from the same gene expression dataset (GSE5281), have an edge overlap of ∼74% (for no edge weight cutoff) and ∼89% (for edge weight ≥0.5); the node overlap always remained 100%. The BC3Net parameters used for performing this analysis are: boot = 100, estimator = “pearson”, disc = “equalwidth”, mtc1 = TRUE, alpha1 = 0.05, adj1 = “bonferroni”, mtc2 = TRUE, alpha2 = 0.05, adj2 = “bonferroni”, weighted = TRUE, igraph = TRUE, verbose = FALSE and number of seed genes = 4808 (see the section on Gathering initial seed genes). However, less frequently appearing, yet plausible, edge interactions could offer the potential for promising candidates that are buried in expression data.

We observed that the intersection between independently generated GRNs saturated after 5–10 repetitions of the BC3Net algorithm on the same dataset. Thus, in order to expand the knowledge space around AD candidates and for completeness, we propose an optimization of the randomness to devise a more recall optimized GRNs. More specifically, we applied the BC3Net algorithm to the same dataset 10 times, named BC3Net10. Finally, we aggregated the 10 independently generated GRNs into one. The final edge weight is now the mean of the computed edge score from 10 GRNs. This increases the prospect of deducing more reasonable functional speculations in complex diseases with the high probability of novelty for further investigations.

### Subnetwork selection and functional enrichment analysis

The choice of a threshold can significantly affect the integrity of the network and the co-expression modules derived from it. In this regard, computed edge weight (mean weight >0.5) from BC3Net10 was used as the filter criteria for selecting significant gene pairs in the generated GRNs. This increases the significance level by 50% for the inferred interactions in each dataset.

**Fig.2:**
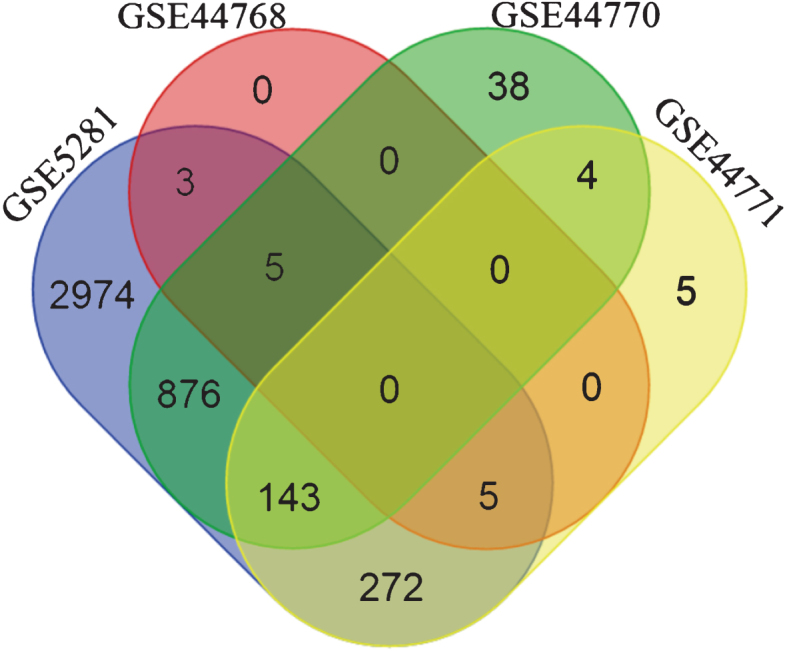
Venn diagram depicting the gene overlap between the subnetworks (edge weight >0.5) of the four datasets, generated using the initial seed. The initial seed was compiled from top 500 genes retrieved by querying SCAIView for Alzheimer’s disease related genes. It is evident that there are no common genes among the four dataset’s subnetworks. Differing factors between platforms, analytical methods, tissue source, etc. could contribute to such a behavior.

The overlap between the inferred interactions/edges was very low (zero genes common to all 4 subnetworks, see Fig. 2) when BC3Net was applied on the initial seed. Several reasons can be presumed for lack of common and stable genes such as different platforms, distinct brain tissues, diverse patient cohort, and treatment heterogeneity. However, numerous studies have already shown that the functional signatures are more stable relative to individual gene level information [45–48]. In this context, to extract the most representative biological pathways for genes in the subnetworks (separately for each dataset), we performed functional enrichment analysis (based on one-sided Fisher’s exact test) for KEGG pathway information using ConsensusPathDB (CPDB) [49] (Release 30). Using the Bioconductor package, *org.Hs.eg.db* [50], we mapped the gene symbols to Entrez gene identifiers obtained from CPDB.

### Identification of enriched candidates and seed gene list enrichment

We devised a strategy to expand the seed through functional enrichment analysis of the individual network modules inferred by the GRNs, enabling us to quantify the saturation of the inferred network. We extracted significant pathways (for the *p*-value <0.05) common between the determined subnetworks of the four datasets, generated using the initial seed. We added a new gene (called enriched candidate) to the seed when the gene belongs to the respective CPDB and KEGG pathway gene set that is significantly enriched across all 4 inferred GRNs and is not present in our initial seed. Further, we repeated the functional enrichment analysis to determine overlapping pathways for the enriched seed. We leveraged the identified enriched candidates in these pathways by subsequent inclusion in the seed iteratively until saturation. Once the seed has reached its saturation, we merge the networks of all iterations, separately for each dataset, to generate an aggregated network. This approach goes beyond just candidate enrichment, corresponding to a maximal AD specificity with minimal noise and harvesting lesser known genes in GRNs.

### Gene list prioritization by genetic variant analysis

For the consensus network, we identified genes (involved in significant pathways and hub genes) to prioritize them using genetic variant analysis. Multiple genetic variants are attributed in the etiology of complex diseases. To investigate the impact of genetic variation, we extracted AD evidences for single-nucleotide polymorphisms (SNPs) from GWAS catalog [51], GWAS Central [52], and gwasDB [53], resulting in 11,314 SNPs. Further, linkage disequilibrium (LD) analysis was carried out to enrich the list of AD associated genetic variants, which were sorted based on their chromosome location. LD is SNP’s property on a contiguous stretch of a chromosome that describes the degree to which an allele of one genetic variant is inherited or correlated with an allele of another genetic variant within a population. The LD analysis was performed using HaploReg v2 (developed by Broad Institute of MIT) [54] based on dbSNP-137 [55], motif instances (based on PWMs provided by the ENCODE project database) [56], enhancer annotations (adding 90 cell types from the Roadmap Epigenome Mapping Consortium) [57], and eQTLs (from the GTex eQTL browser) [58]. With LD threshold cutoff of r2 = 0.8, we obtained 115,782 SNPs. Further on, these SNPs were filtered based on the ENSEMBL SNP Effect predictor that estimates the influence of SNP variants on the respective transcripts of a gene and their gene products [59], shortlisting 4,831 SNPs. Genes obtained from the aggregated networks were boiled down to those associated with shortlisted SNPs. Finally, these refined genes were ranked using a cumulative score of their SNPs from RegulomeDB [60], dbSNP’s functional annotation [55], ENSEMBL’s Variant Effect Predictor [61], and regulatory feature annotation by ENSEMBL variant database [62]. RegulomeDB’s ranking is based on the functional annotations from ENCODE database [63], chromatin states from the Roadmap Epigenome Consortium [57], DNase-footprinting [64], position weighted matrix for transcription factor binding [65], and DNA methylation [66].

## RESULTS AND DISCUSSION

### Algorithm convergence and network properties

Under the premise that lesser known genes are not prominently represented in literature, we extended the set of seed genes through functional enrichment (see the section on Subnetwork selection and functional enrichment analysis). As depicted in Fig. 1, BC3Net10 was applied on the identified four datasets for different seed lists to generate AD GRNs. Iteration 1, where we generated GRNs for SCAIView genes, resulted in 10 overlapping and significant pathways between the four datasets. From these pathways, we obtained 820 genes that were earlier not present in the seed. Hence, there is a clear need for further enrichment of the seed, which is done by including these newly identified candidates to the seed and repeating the functional enrichment step. In the second iteration, we identified 38 overlapping significant pathways between the datasets. This iteration continues seven times until there are no newer candidates to be added. Table 2 provides the statistics of the number of pathways identified in each iteration, along with the number of enriched candidate genes that were added to the seed. A detailed list of the enriched candidates (as HGNC symbols) identified in each iteration is provided in Supplementary File 1. A sharp increase in the number of enriched candidates is observed in the first two iterations, which drops to zero in the seventh iteration. We assume that this indicates the completeness of the gene set that belongs to AD, specific to the selected four datasets.

**Table 2:** Statistics of the iterative functional enrichment approach

Iteration (i)	Seed	No. of overlapping	No. of enriched candidate
		pathways between the	genes obtained from the
		four datasets	overlapping pathways
1	SCAIView (500)	10	820
2	i1+820	38	1148
3	i2+1148	30	361
4	i3+361	30	84
5	i4+84	32	41
6	i5+41	33	7
7	i6+7	37	–

**Fig.3:**
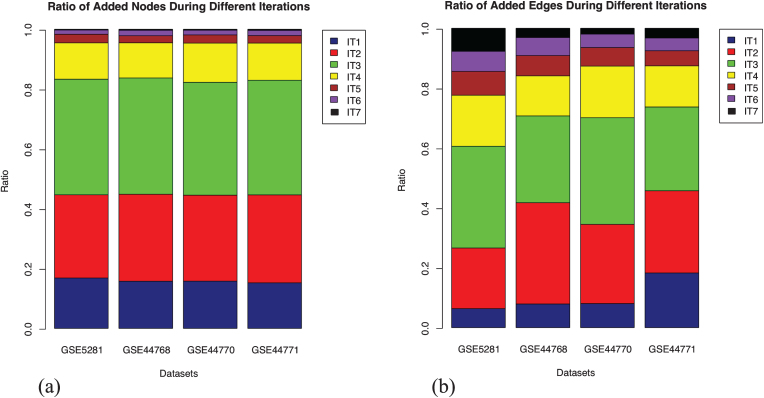
Stratification of the nodes and edges in four aggregated networks. Each stack in the bar plot represents the fraction of nodes added in that iteration (IT) relative to the aggregated network (considered as 1). The addition of nodes remained stable across the datasets in each iteration. However, the inclusion of edges varies, which could be presumed due to newly inferred interactions from the newly included nodes in each iteration. (a) Fraction of added nodes in different iterations; (b) Fraction of added edges in different iterations.

Extension of the GRNs using enriched seed is a knowledge guided approach, which relies on the functional information derived from the gene expression data. We note that the GRNs grow progressively, both inferred interactions and the participating candidates, but in the process, eliminates few of the previously inferred interactions. A potential reason is that the extension of the expression matrix with new seed contributes to a shift in the significance of the inferred interactions by BC3Net. It implies that although we obtain a final GRN for saturated seed (iteration 7), aggregating networks from earlier iterations could capture interactions that were previously inferred as potential. The fraction of nodes and edges from each iteration that makes up the aggregated network, for each dataset, is presented in Fig. 3. We observe that the addition of nodes, in each iteration, across datasets, remained stable whereas the same cannot be said for the edges. The variance in edges could be presumed that the newly added set of genes bring in higher functional relevance through newly inferred interactions in one or the other iteration.

**Fig.4:**
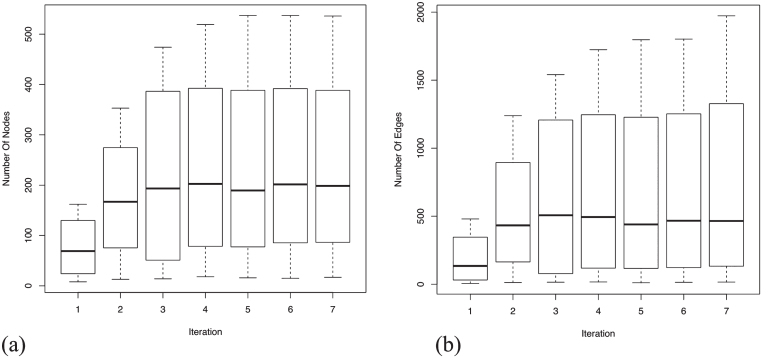
Mean and variance distribution across four datasets for the added nodes and edges in each iteration. Enrichment of nodes and edges reach saturation after 7th iteration, suggesting the completeness of the generated GRNs. Relatively high number of edges (see y-axis range) show immense inter-connectivity between the genes in the GRNs. (a) Boxplot for mean and variance distribution of nodes; (b) Boxplot for mean and variance distribution of edges.

An assessment of the completeness of a GRN for AD specific genes can be precisely estimated by plotting the mean and the variance of the number of nodes and edges present in each dataset for each iteration. From Fig. 4a, it is evident that the enrichment of the most relevant genes reach saturation. This increases the statistical significance of the GRNs suggesting an increment in the biological confidence. It is apparent that not all the genes present in the seed agree across platforms due to various differing experimental factors. However, we expect functional signatures across the datasets to be more agreeable. Analyzing edges (see Fig. 4b), we observe that they orient three times, at saturation, to the number of nodes. The relative higher number of edges demonstrate that the gene sets are highly related, showing immense inter-connectivity between several functional modules. The high variance observed, in both nodes and edges, is contributed by the large network size of GSE5281 relative to the other three datasets. Details of the number of nodes and edges present in each dataset at each iteration is provided are Supplementary File 2.

### Hub genes

Hub genes have a higher grade of lethality when dysregulated in a pathological condition, referred to as centrality lethality rule [67]. For each aggregated GRN, a gene was defined as a hub gene when it had a higher degree of distribution (>95% quantile). By this criterion, we identified 29 in GSE5281, 8 in GSE44768, 14 in GSE44770, and 1 in GSE44771 as hub genes. Table 3 displays the list of identified hub genes along with their node degree and pathway annotation (only for significant pathways, see Functional homogeneity across datasets section). Interestingly, there were no common hub genes between the four datasets. It was evident that six of the hub genes were perturbed in multiple pathways. Many of the hub genes were functionally enriched in neurotrophin signaling, endocytosis, and estrogen signaling pathways. Additional associated pathways with hub genes include calcium signaling, adipocytokine signaling, NOD-like receptor signaling, insulin signaling, apoptosis, thyroid signaling, and pancreatic secretion. The majority of these hub genes formed a connected subnetwork within each dataset, indicative of a possible cooperative effect in AD pathology (see Supplementary Figure 1). In the case of GSE44771, due to the presence of a single hub gene, we extracted the largest subnetwork associated with HSPA2.

**Table 3:** Hub genes identified in the aggregated network for the four datasets. The genes are sorted by their hub degree within each dataset. Only significant pathways are listed here (see Table 4 for the list)

GEO ID	Gene Symbols	Hub Degree	Pathway Annotation (CPDB)	Similar results in
				other datasets?
GSE5281	HFE	244	–	–
	ATP2A3	162	Calcium signaling, pancreatic	–
			secretion
	GLP1R	150	Insulin Secretion	–
	ADRBK1	145	Endocytosis	GSE44770
	CACNG4, CACNG6	141	–	–
	KCNJ5	132	Estrogen signaling	–
	P2RX2	130	Calcium signaling	GSE44770
	KPNA2	122	–	–
	NOX1	118	–	–
	CACNG5	113	–	–
	EPN1	113	Endocytosis	–
	WAS	112	–	–
	CASP10	111	Apoptosis	–
	HSPB6, EPHA4	109	–	–
	ADNP	108	–	–
	DNAH3	106	–	–
	GRIN2A	105	Calcium signaling	–
	UBQLN1	101	–	–
	IL34, ATP5A1, UBE2L3	100	–	–
	DPYSL2	99	–	–
	FOLR2	98	Endocytosis	–
	NPR1	96	–	–
	DNM1L, KLC1, ATP5G3	92	–	–
GSE44768	RASGRF1	80	–	–
	DNAL4	63	–	–
	EPHA1	60	–	–
	CHRND	59	–	–
	TRPC1	54	Pancreatic secretion	GSE5281, GSE44770
	PAK7	50	–	–
	NDUFA4	44	–	–
	CHMP4B	44	Endocytosis	–
GSE44770	IVNS1ABP	103	–	–
	FGF18	92	–	–
	ATF2	90	Estrogen signaling, Insulin secretion	–
	CTSG	88	–	–
	GABRE	86	–	–
	FBXL2	81	–	–
	GAPDH	75	–	–
	DIO1	72	Thyroid hormone signaling	–
	CACNB3, CDK2	66	–	–
	NFKBIB	66	Adipocytokine signaling,	GSE44768
			neurotrophin signaling, NOD-like
			receptor signaling
	PRDM4	64	Neurotrophin signaling	–
	MAPK9	63	Adipocytokine signaling,	–
			neurotrophin signaling, NOD-like
			receptor signaling
	PIK3CB	63	Apoptosis, estrogen signaling,	GSE5281
			neurotrophin signaling, thyroid
			hormone signaling
GSE44771	HSPA2	18	Endocytosis, estrogen signaling	–

### Functional homogeneity across datasets

Are the core functional modules (set of interconnected-genes) unique to a human brain region or do they depict patterns reflecting the tight linkage between different regions of the brain? To address these questions, we compared the final determined significant pathways across the four aggregated GRNs (outlined in Methods). The functional enrichment analysis revealed 187 in GSE5281, 120 in GSE44768, 170 in GSE44770, and 43 in GSE44771 inferred modules within significant KEGG pathways. We computed a simple overlap between the four GRNs to assess the conserved pathways, resulting in 34 pathways. Because this list contained pathways that were not directly relevant to the core pathophysiology of AD, we categorized them into subsets based on their pertinence to AD, see Table 4. Please refer to Supplementary File 4 for details of summary statistics. From these, we chose to focus on pathways that exacerbate the AD phenotype, classified as “Potential”. Table 4 also provides the statistics of the number of genes enriched for these pathways in each dataset. Interestingly, there are no common genes between the four datasets when compared at the pathway level. However, many of the genes are shown to be involved in more than one potential pathway, providing the basis for functional connectivity in AD.

**Table 4:** Landscape of significant pathways (*p*-value <0.05) determined across datasets

Common	Pathway Category	Total no. of genes	Number of genes enriched for the pathway				
Pathways		in the pathway	GSE5281	GSE44768	GSE44770	GSE44771	Consensus
Cancer	Basal cell carcinoma	55	5	2	7	1	15
Cancer	Colorectal cancer	62	6	2	8	1	14
Cancer	Pathways in cancer	398	64	27	40	3	119
Cancer	Small cell lung cancer	86	15	4	9	2	27
Comorbidity	Amyotrophic lateral sclerosis	51	14	2	5	1	18
Comorbidity	Arrhythmogenic right ventricular cardiomyopathy	74	13	5	8	2	21
Comorbidity	Dilated cardiomyopathy	90	17	6	7	2	26
Comorbidity	Hypertrophic cardiomyopathy	83	14	7	7	2	23
Comorbidity	Rheumatoid arthritis	91	10	4	7	1	20
Infection	Epithelial cell signaling in Helicobacter pylori infection	68	9	2	6	1	16
Infection	Influenza A	177	25	4	22	2	46
Infection	Shigellosis	61	12	3	7	1	19
Infection	Toxoplasmosis	120	14	3	12	2	26
Infection	Tuberculosis	179	21	4	23	3	46
Infection	Vibrio cholera infection	54	9	2	2	1	13
Infection	Viral myocarditis	60	12	2	5	2	19
Others	Melanogenesis	101	18	3	10	1	30
Others	Neuroactive ligand-receptor interaction	275	57	24	30	3	98
Potential	Apoptosis	86	14	2	6	1	20
Potential	Calcium signaling pathway	180	43	12	16	2	62
Potential	Endocytosis	213	47	10	21	4	70
Potential	Neurotrophin signaling pathway	120	24	6	17	1	44
Potential	NOD-like receptor signaling pathway	57	9	3	6	1	16
Potential	PPAR signaling pathway	69	11	4	9	2	22
Potential	Synaptic vesicle cycle	63	15	4	8	1	26
Potential	Adipocytokine signaling pathway	70	17	6	8	1	27
Potential	Insulin secretion	86	18	3	10	1	28
Potential	Pancreatic secretion	96	21	5	9	1	30
Potential (hormones)	Estrogen signaling pathway	100	23	4	7	1	32
Potential (hormones)	Thyroid hormone signaling pathway	119	26	3	10	1	37
Potential (others)	Lysosome	122	13	7	11	4	33
Potential (others)	Phagosome	155	31	4	16	2	48

### Regulatory underpinning across Consensus network

**Fig.5:**
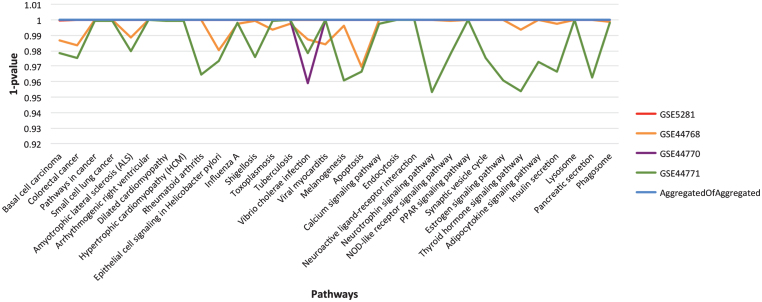
The landscape of *p*-value for the final list of significant pathways. For easy visualization, we have used 1-*p* value instead of *p*-value on Y-axis. Each line in the graph represents aggregated GRN for specified dataset (see chart legend). The listed pathways show higher significance level in consensus GRN in comparison to the individual dataset aggregated GRNs.

As described in the section on Functional homogeneity across datasets, the genes in different GRNs are complementary for the top significant pathways. Thus, to provide a broader coverage than a single GRN and to infer stronger relationships through consensus, we merged the four aggregated GRNs into one, called consensus network. What we expect is to uplift the most promising pathways due to the assembly of more participating genes. To assess the concept of functional enrichment, we plot the *p*-values of all the significant pathways, listed in Table 4, for each of the aggregated and consensus GRNs, see Fig. 5. From the figure, it is evident that these pathways have attained higher significance level (better *p*-values) in consensus GRN due to the gene complementarity from the aggregated GRNs.

### Prioritizing through genetic variant analysis

We compiled 608 genes from listed significant pathways across datasets (see Table 4) and hub genes. We mapped these genes to the 4,831 shortlisted ENSEMBL SNPs (see Methods). For the obtained 167 mapped genes, we ranked them based on the calculated cumulative score for their potential functional consequences in a disease context. Restricting the ranked genes to the RegulomeDB score of 3, we generated a final list of 44 high ranked genes. In addition, we looked into the AD GWAS meta-analysis study carried out by Lambert et al. [68]. Among all their listed genes carrying genetic AD risks, we found three (AP2A2, DPYSL2, and EPHA1) of them to be present in our 608 gene list, including one (EPHA1) newly reported in their study; these three were added to our final gene list. Please refer to Table 5 for detailed ranking and RegulomeDB score. Additional investigation revealed 14 out of 47 genes from our final gene list are either validated by eQTLs studies or experimentally evident that the SNPs are linked to the active promoter region of the gene. These genes include IL1B, NSF, HLA-F, NOTCH4, VCL, PSAP, STX2, GGA2, STK11, CSF3R, LMNA, CTNNA2, HLA-C, and RAB11FIP4. When we performed a comprehensive analysis of the biomedical literature, we found that many of these genes had no evidence of being linked to AD, but were rather known to be involved in AD co-morbidity diseases (see Supplementary Table 1).

**Table 5:** List of genes prioritized using genetic variant analysis

Rank	Gene	RegulomeDB	No. evidences	Pathways involved
	Symbol	score	for AD
1	IL1B	1b	1073	Apoptosis, NOD-like receptor signaling
2	NSF	1d	8	Synaptic vesicle cycle
3	HLA-F	1f	0	Endocytosis
4	NOTCH4	1f	3	Thyroid hormone signaling
5	VCL	1f	10	Shigellosis
6	PSAP	1f	3	Lysosome
7	STX2	1f	2	Synaptic vesicle cycle
8	GGA2	1f	4	Lysosome
9	STK11	1f	7	Adipocytokine signaling
10	CSF3R	1f	5	Pathways in cancer
11	LMNA	1f	11	Arrhythmogenic right ventricular cardiomyopathy, Dilated cardiomyopathy, Hypertrophic cardiomyopathy
12	CTNNA2	1f	3	Arrhythmogenic right ventricular cardiomyopathy
13	HLA-C	1f	1	Endocytosis
14	RAB11FIP4	1f	0	Endocytosis
15	GRIN2A	2a	52	Calcium signaling
16	RBX1	2a	0	Viral Myocarditis
17	KCNJ5	2a	0	Estrogen signaling
18	EPHA4	2b	18	Hub Genes
19	CACNG4	2b	0	Arrhythmogenic right ventricular cardiomyopathy, Dilated cardiomyopathy, Hypertrophic cardiomyopathy
20	PLA2G5	2b	7	Pancreatic secretion
21	ATP2B4	2b	1	Calcium signaling, pancreatic secretion
22	P2RY14	2b	0	Neuroactive ligand receptor interaction
23	P2RY13	2b	0	Neuroactive ligand receptor interaction
24	PTGER4	2b	11	Neuroactive ligand receptor interaction
25	ARAP3	2b	0	Endocytosis
26	FGF1	2b	22	Pathways in cancer
27	RPS6KA2	2b	0	Neurotrophin signaling
28	RAPGEF1	2b	0	Neurotrophin signaling
29	GABBR2	2b	1	Estrogen signaling
30	PRF1	2b	1	Viral myocarditis
31	ITGA8	2b	0	Arrhythmogenic right ventricular cardiomyopathy, Dilated cardiomyopathy, Hypertrophic cardiomyopathy
32	AP2A2	2b	0	Endocytosis, Synaptic vesicle cycle
33	ITPR2	2b	2	Calcium signaling, Estrogen signaling, pancreatic secretion
34	MED13L	2b	0	Thyroid hormone signaling
35	COL4A1	2b	0	Pathways in cancer
36	KCNJ6	2b	3	Estrogen signaling
37	ATP2A3	2b	0	Calcium signaling, Pancreatic secretion
38	ASAP2	3a	1	Endocytosis
39	FYN	3a	70	Viral myocarditis
40	NTRK2	3a	124	Neurotrophin signaling
41	PAK1	3a	7	Epithelial cell signaling in Helicobacter pylori infection
42	COL4A2	3a	0	Small cell lung cancer, Pathways in cancer
43	BMP4	3a	5	Thyroid hormone signaling
44	GABRB3	3a	0	Neuroactive ligand receptor interaction
45	CEBPB	3a	12	Tuberculosis
46	EPHA1	5	31	Hub Genes
47	DPYSL2	5	47	Hub Genes

### Well known prioritized AD candidates

Apart from the new novel candidates, our method also determined well-known candidates (nearly 50 articles in AD) such as IL1B, NTRK2, GRIN2A, FYN, and DPYSL2. The IL1B gene is a pro-inflammatory cytokine that has been long studied for its modulatory effect in AD. It is reported that the expression of IL1B significantly increases with the increase of AD-related neurofibrillary pathology [69]. Synaptic plasticity, such as long-term potentiation, is crucial for learning and memory. A neurotransmitter modulator, BDNF, mediates neuronal survival and plasticity by regulating neurotrophins through NTRK2. AD patients with cognitive deficits have been accounted with reduced levels of BDNF [70–72]. Similarly, GRIN2A is a subunit of NMDA receptors, whose reduced expression increases the vulnerability of neurons to excitotoxicity in AD, correlated with cognitive impairment due to reduced plasticity [73, 74]. A strong correlation between lower levels of BDNF and cognitive deficits in AD patients was recently reported by Buchman et al. [75]. Recent research work has suggested BDNF as an upstream regulator of FYN gene, a Src family kinase, leading to enhanced cascade effect of NMDA mediated excitotoxicity and regulates the activity of hyperphosphorylated tau [76, 77]. In addition, it mediates the synaptic deficits that are induced by Aβ [78]. DPYSL2 mediates synaptic signaling to facilitate neuronal guidance through regulation of calcium channels. Furthermore, FYN phosphorylates DPYSL2 within the brain and its hyperphosphorylation is causally related to Aβ neurotoxicity [79]. Taken together, these findings suggest that synaptic transmission is critical for regulating Aβ production in AD. Further studies, along these lines, may provide insights into the precise molecular mechanism underlying this part of AD etiology.

### Mechanistic interpretation of newly prioritized candidates in neurotransmission

Neurotransmission is a pivotal brain function that declines with progressing age. However, in the case of AD, there is a drastic and non-uniform deterioration of synaptic neurotransmission [80]. It is known that soluble oligomeric Aβ, rather than insoluble deposits that form plaques (extracellular), are detrimental to synaptic currents through calcium channel modulation, leading to excitotoxic cascades that mediate AD progression [81] and are related to the formation of neurofibrillary tangles (intracellular) [82]. Emerging research strongly supports the hypothesis of dysregulated calcium homeostasis influencing the presence of neurotoxic Aβ in AD patients [83]. Increased endocytosis activity, enlarged endosomes, has been reported by Cataldo et al. [84] as the earliest intraneuronal neuropathologic feature of AD, subsequently impairing the modulation of NMDA receptor. NMDA excitotoxicity leads to the pathological overload of calcium resulting in synaptic impairment and ultimately neuronal death [85].

**Fig.6:**
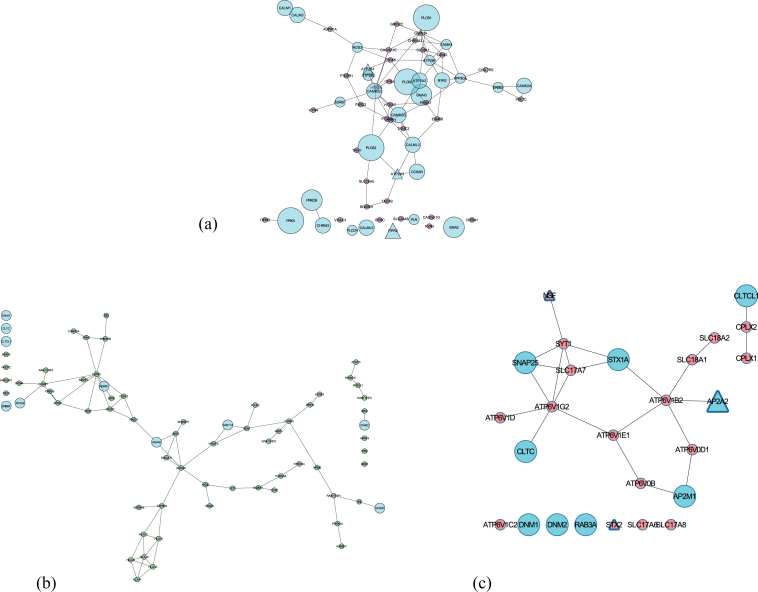
Subnetworks of the three shortlisted potential pathways (extracted from consensus network) involved in neurotransmission. Nodes in Cyan are involved in more than one pathways and the size of the nodes depends on the number of pathways involved. Triangle nodes represent the presence of a SNP. (a) Calcium signaling pathway; (b) Endocytosis pathway; (c) Synaptic vesicle cycle.

We observed that three of the “Potential” pathways are significantly involved in neurotransmission: calcium signaling, endocytosis, and synaptic vesicle cycle (see Fig. 6). To assess the modularity of the prioritized candidates in these identified pathways, we extracted the functional relevance of their combination. This confirms our previous findings associated with well-known candidates (see Section Well known prioritized AD candidates). To gain new insights in this context we focused on lesser known prioritized candidates in AD that are involved in these three pathways (less than 5 publications): STX2, HLA-F, HLA-C, RAB11FIP4, ARAP3, AP2A2, ATP2B4, ATP2A3, and ITPR2. Below, we briefly discuss the possibility of these candidates to presumably bear potential as new targets in AD (detailed description is provided in Supplementary File 5).

The presence of Aβ oligomers impairs the process of STX2 binding to SNARE proteins hindering the effective release of neurotransmitter during synaptic vesicle fusion in the presence of increased calcium influx [86, 87]. From several previous studies, one can postulate that HLA-F and HLA-C mediated dysregulated trafficking of amyloid plaques in endocytosis could be correlated to the memory deficits in early AD [88–90]. Several recent evidence point to the fact that faulty Aβ processing can be detected in the membrane trafficking events (linked to RAB11 proteins) of early endosomes, promoting an effective early diagnosis [91, 92]. ARAP3 modulates actin cytoskeleton’s remodeling by regulating ARF and RHO family members [93] and a growing body of evidence suggest that axonal transport defects due to its abnormality could be responsible for neurite degeneration and tau toxicity [94–97]. Impairment of APP shuttling by AP2A2 (part of AP-2 complex [98]) from the endocytotic pathway to autophagy degradation leads to intracellular aggregation of Aβ [99]. The next three candidates (ATP2B4, ATP2A3, and ITPR2) participate in neuronal calcium shuttling. A substantial body of evidence indicates ATP2B4, a plasma membrane Ca(2+) ATPases (PMCAs) is inhibited by Aβ peptides [100], causing cell death [101]. Similarly, ATP2A3’s function in handling calcium load and release is perturbed by the mutation in PSEN1 (regulates the intramembrane Aβ processing) [102]. Increased expression of ITPR2 could lead to calcium toxicity in neurons and finally cell death [103, 104].

### Conclusion

The identification of biological mechanisms underlying normal physiology and— when dysregulated— contributing to or even directly causing disease phenotypes is a key objective of current integrative biology. Strategies, both data- and knowledge-driven, for mechanism-identification have shown to deliver valuable insights into disease mechanisms; however, both approaches have their specific drawbacks. Here, we demonstrate a new approach that combines literature-based knowledge and data-driven analysis through gene regulatory networks in a flexible and adaptive way. Thus, allowing us to identify stable and robust patterns of co-expressed genes across several large disparate datasets, in parallel, which enhances the interpretability around “interesting patterns” of co-regulated genes.

We developed an adapted version of BC3Net, called as BC3Net10, that supports a more fine-granular specification of functional context by “injecting” sets of seed genes (derived from literature) into the algorithm. The seed genes were iteratively extended through functional enrichment applied on generated GRNs until convergence. Through several iterations of “selecting and injecting seed genes” and subsequent co-expression analysis, we come up with stable, knowledge-instructed GRNs across several experiments. We show the ability of our approach to identify functional context around subtle signals that would typically be expected for highly individual “modifier” functions not in the core of a dysregulation event, but have the potential to modulate the clinical path of a disease. Hence, making this approach ideally suited for biomarker identification. We show that by the enhanced functional interpretation of the GRNs shed more light on the role of neurotransmission physiology in early dysregulation events presumed to be part of AD etiology. This warrant further investigation of their potential as therapeutic targets.

We would like to point out that there is more potential to the method presented here: in the course of IMI-project AETIONOMY we found limited coverage of signals in knowledge based models coming from the analysis of either gene expression or genetic variation information (GWAS studies). The methodology presented here bears the potential to establish biologically meaningful context around “isolated signals” in knowledge-based models to “embed” previously “non-interpretable” (at functional level) genes into a wider (knowledge based) context. Insights drawn from this approach could provide a novel foundation for the formation of new hypotheses. Although microarray data is the obvious starting point, the next logical step would be to extend this work to incorporate orthogonal datatypes such as NGS and single cell data. This could provide a broader view of disease etiology and enable comprehensive in silico investigations. It remains to be shown that the method we introduce here scales up to a really large number of experiments of different sample size.

## DISCLOSURE STATEMENT

The current affiliation of Dr. Philipp Senger is CLS Head of Translational R&D, Bayer CropScience, Alfred-Nobel-Straße 50, 40789 Monheim, Germany. The current affiliation of Mufassra Naz is Institute of Business & Information Technology, University of Punjab, Lahore. Both contributed to this work during their employment at Fraunhofer SCAI.

Authors’ disclosures available online (http://j-alz.com/manuscript-disclosures/17-0011r2).

## Supplementary Material

Supplementary File 1

Supplementary File 2

Supplementary File 3

Supplementary File 4

Supplementary File 5

Supplementary Table 1

Supplementary Figure 1
