# Supplementary material for: Analytical Strategy to Prioritize Alzheimer’s Disease Candidate Genes in Gene Regulatory Networks Using Public Expression Data
Source: J Alzheimers Dis. 2017 Aug 14;59(4):1237–54. doi: 10.3233/JAD-170011 (PMC5611835; doi:10.3233/JAD-170011)
Supplement: Supplementary File 5 [file jad-59-jad170011-s005.doc]

Detailed mechanistic information of the newly prioritized candidates in AD is provided below.

**STX2 mediates neurotransmission**

Among the identified potential candidates, STX2 belongs to syntaxin protein family and binds to SNARE proteins that mediates effective neurotransmitter release during synaptic vesicle fusion in the presence of increased calcium influx [1,2]. Reduced formation of SNARE complex assembly was observed in the postmortem brains of AD patients [3]. A recent study by Yang et al. [4] showed that Aβ oligomers impair SNARE-mediated exocytosis in mice that hinders synaptic vesicle docking, contributing to cognitive impairment. Thus, inhibiting Aβ oligomers from binding to syntaxin proteins could help restore the SNARE functionality and improve the cognitive ability of the early AD patients.

**HLA-F and HLA-C involved in amyloid-β trafficking**

Microglia are resident immune response cells and when activated upregulates surface receptors such as class I major histocompatibility complex (MHC), which includes HLA-F and HLA-C proteins [5]. It has been reported that the increased frequency of MHC class 1 proteins is observed in AD patients [6]. Additionally, studies suggest that pro-inflammatory responses due to extracellular Aβ deposits are involved in worsening the cognitive decline in AD patients [7]. One can postulate that MHC class I mediated dysregulated trafficking of amyloid plaques in endocytosis could be correlated to the memory deficits in early AD [6–8].

**RAB11FIP4 as modulator of neurotransmission**

The next candidate gene, RAB11FIP4, a part of GTPase family of Rab11 proteins, is associated with endosomal recycling and participates in polarized neurite growth [9–11]. RAB11FIP4 gene consists of a EF-hand calcium binding motif that regulates Ca (2+) in exocytosis. Recently, Chutna et al. elucidated its role in mediating α synuclein aggregation and toxicity in Parkinson’s disease [12]. Much of the recent research have suggested its role as a neurotransmission modulator whose dysregulation could inhibit vesicle tethering with SNARE proteins, implicated in synaptic and cognitive deficits, in several neurodegenerative diseases [13,14]. In AD, several recent evidence point to the fact that faulty amyloid-β processing can be detected in the membrane trafficking events (linked to RAB11 proteins) of early endosomes, promoting an effective early diagnosis [15,16]. Precisely, Zhao et al. demonstrated that reduced expression of PICALM (whose genetic variant is implicated in AD), in humans and mice, guides the dysregulation of PICALM/clathrin–dependent internalization of the Aβ-LRP1 complex by RAB11, affecting the Aβ traffic in endothelial transcytosis and its clearance [17].

**ARAP3 regulates actin cytoskeleton stability**

The actin cytoskeleton plays a key role in synaptic activity and ARAP3 modulates its remodeling by regulating ARF and RHO family members [18]. A growing body of evidence suggest that axonal transport defects due to abnormalities in actin cytoskeleton could be responsible for neurite degeneration and tau toxicity by interfering with mitochondrial dynamics in neurons [19–22]. Since much of the research is focused on Aβ plaques and neurofibrillary tangles, actin aggregates, although observed in human AD brains, are less appreciated [23–25]. At this time, however, more research is needed to understand the role of ARAP3 in AD.

**AP2A2 internalizes APP and BACE1 proteins**

Protein AP2A2 is part of adapter protein complex 2 (AP-2) that serves as a cargo receptor for internalization of membrane proteins in clathrin-mediated endocytosis and recycling of synaptic membrane [26]. Recent mouse model evidences in AD, report the involvement of AP-2 in APP and BACE1 internalization [27,28]. Within neurons, and glial cells, increased expression of PICALM can affect the role of AP-2 clathrin-mediated endocytotic clearance of Aβ [29]. Impairment of APP shuttling by AP-2 from endocytotic pathway to autophagy degradation leads to intracellular aggregation of Aβ [30].

**ATP2B4, ATP2A3, and ITPR2 maintains calcium homeostasis in neuron**

The next three candidates (ATP2B4, ATP2A3, and ITPR2) participate in neuronal calcium shuttling. A substantial body of evidence indicates ATP2B4, a plasma membrane Ca (2+) ATPases (PMCAs), regulated by calmodulin, critically maintain calcium homeostasis of the neuron [31]. PMCAs is the only calcium pump in the brain, which is inhibited by the presence of Aβ peptides [32]. This inhibition leads to the failure of maintaining the intracellular concentration of Ca(2+), causing cell death [33]. Similarly, ATP2A3 encodes SERCA Ca (2+)-ATPases (SERCA) that are intracellular calcium pumps in the endoplasmic reticulum. PSEN1 is a principal component of the γ-secretase that regulates intramembrane Aβ processing, whose mutation could perturb with SERCA function in handling calcium load and release [34]. Enhancing PSEN1 levels could accelerate the clearance of calcium through secondary messenger ITPR2. ITPR2 has been identified to play a pivotal role in maintaining and release of intracellular Ca (2+) stores. However, increased expression of ITPR2 could lead to calcium toxicity in neurons and finally cell death [35,36].

**REFERENCES**

[1] Ghiani CA, Starcevic M, Rodriguez-Fernandez I a, Nazarian R, Cheli VT, Chan LN, Malvar JS, de Vellis J, Sabatti C, Dell’Angelica EC (2010) The dysbindin-containing complex (BLOC-1) in brain: developmental regulation, interaction with SNARE proteins and role in neurite outgrowth. *Mol Psychiatry* **15**, 115, 204–215.

[2] Band AM, Kuismanen E (2005) Localization of plasma membrane t-SNAREs syntaxin 2 and 3 in intracellular compartments. *BMC Cell Biol* **6**, 26.

[3] Sharma M, Burre J, Sudhof TC (2012) Proteasome inhibition alleviates SNARE-dependent neurodegeneration. *Sci Transl Med* **4**, 147ra113-147ra113.

[4] Yang Y, Kim J, Kim HY, Ryoo N, Lee S, Kim Y, Rhim H, Shin YK (2015) Amyloid-β oligomers may impair SNARE-mediated exocytosis by direct binding to syntaxin 1a. *Cell Rep* **12**, 1244–1251.

[5] Wojtera M, Sobow T, Kloszewska I, Liberski PP, Brown DR, Sikorska B (2012) Expression of immunohistochemical markers on microglia in Creutzfeldt-Jakob disease and Alzheimer’s disease: morphometric study and review of the literature. *Folia Neuropathol* **50**, 74–84.

[6] De Groot CJA, Hulshof S, Hoozemans JJM, Veerhuis R (2001) Establishment of microglial cell cultures derived from postmortem human adult brain tissue: Immunophenotypical and functional characterization. *Microsc Res Tech* **54**, 34–39.

[7] McGeer PL, McGeer EG (2013) The amyloid cascade-inflammatory hypothesis of Alzheimer disease: implications for therapy. *Acta Neuropathol* **126**, 479–497.

[8] Nelson PA, Sage JR, Wood SC, Davenport CM, Anagnostaras SG, Boulanger LM (2013) MHC class I immune proteins are critical for hippocampus-dependent memory and gate NMDAR-dependent hippocampal long-term depression. *Learn Mem* **20**, 505–517.

[9] Wallace DM, Lindsay AJ, Hendrick AG, McCaffrey MW (2002) The novel Rab11-FIP/Rip/RCP family of proteins displays extensive homo- and hetero-interacting abilities. *Biochem Biophys Res Commun* **292**, 909–15.

[10] Ng EL, Tang BL (2008) Rab GTPases and their roles in brain neurons and glia. *Brain Res Rev* **58**, 236–246.

[11] Greenfield JP, Leung LW, Cai D, Kaasik K, Gross RS, Rodriguez-Boulan E, Greengard P, Xu H (2002) Estrogen lowers alzheimer β-amyloid generation by stimulating trans-Golgi network vesicle biogenesis. *J Biol Chem* **277**, 12128–12136.

[12] Chutna O, Gonçalves S, Villar-Piqué A, Guerreiro P, Marijanovic Z, Mendes T, Ramalho J, Emmanouilidou E, Ventura S, Klucken J, Barral DC, Giorgini F, Vekrellis K, Outeiro TF (2014) The small GTPase Rab11 co-localizes with α-synuclein in intracellular inclusions and modulates its aggregation, secretion and toxicity. *Hum Mol Genet* **23**, 6732–6745.

[13] Giorgini F, Steinert JR (2013) Rab11 as a modulator of synaptic transmission. *Commun Integr Biol* **6**, 10–12.

[14] Osaka M, Ito D, Yagi T, Nihei Y, Suzuki N (2015) Evidence of a link between ubiquilin 2 and optineurin in amyotrophic lateral sclerosis. *Hum Mol Genet* **24**, 1617–1629.

[15] Thompson AJ, Williamson R, Schofield E, Stephenson J, Hanger D, Anderton B (2009) Quantitation of glycogen synthase kinase-3 sensitive proteins in neuronal membrane rafts. *Proteomics* **9**, 3022–3035.

[16] Chia PZC, Toh WH, Sharples R, Gasnereau I, Hill AF, Gleeson PA (2013) Intracellular itinerary of internalised β-secretase, BACE1, and its potential impact on β-amyloid peptide biogenesis. *Traffic* **14**, 997–1013.

[17] Zhao Z, Sagare AP, Ma Q, Halliday MR, Kong P, Kisler K, Winkler EA, Ramanathan A, Kanekiyo T, Bu G, Owens NC, Rege S V, Si G, Ahuja A, Zhu D, Miller CA, Schneider JA, Maeda M, Maeda T, Sugawara T, Ichida JK, Zlokovic BV (2015) Central role for PICALM in amyloid-β blood-brain barrier transcytosis and clearance. *Nat Neurosci* **18**, 978–987.

[18] Porat-Shliom N, Milberg O, Masedunskas A, Weigert R (2013) Multiple roles for the actin cytoskeleton during regulated exocytosis. *Cell Mol Life Sci* **70**, 2099–2121.

[19] De Vos KJ, Grierson AJ, Ackerley S, Miller CCJ (2008) Role of axonal transport in neurodegenerative diseases. *Annu Rev Neurosci* **31**, 151–173.

[20] DuBoff B, Götz J, Feany MB (2012) Tau promotes neurodegeneration via DRP1 mislocalization in vivo. *Neuron* **75**, 618–632.

[21] Fulga TA, Elson-Schwab I, Khurana V, Steinhilb ML, Spires TL, Hyman BT, Feany MB (2007) Abnormal bundling and accumulation of F-actin mediates tau-induced neuronal degeneration in vivo. *Nat. Cell Biol* **9**, 139–148.

[22] Halstead JM, Lionnet T, Wilbertz JH, Wippich F, Ephrussi A, Singer RH, Chao JA (2015) Translation. An RNA biosensor for imaging the first round of translation from single cells to living animals. *Science* **347**, 1367–671.

[23] Lazarov O, Morfini GA, Pigino G, Gadadhar A, Chen X, Robinson J, Ho H, Brady ST, Sisodia SS (2007) Impairments in fast axonal transport and motor neuron deficits in transgenic mice expressing familial Alzheimer’s disease-linked mutant presenilin 1. *J Neurosci* **27**, 7011–7020.

[24] Stokin GB (2005) Axonopathy and transport deficits early in the pathogenesis of Alzheimer’s disease. *Science* **307**, 1282–1288.

[25] Yao J Khan AN (2012) Involvement of actin pathology in Alzheimer’s disease. *Cell Dev. Biol.* **2**, 2–4.

[26] UniProt Database.

[27] Poulsen E, Larsen A, Zollo A, Jørgensen A, Sanggaard K, Enghild J, Matrone C (2015) New insights to clathrin and adaptor protein 2 for the design and development of therapeutic strategies. *Int J Mol Sci* **16**, 29446–29453.

[28] Maesako M, Uemura M, Tashiro Y, Sasaki K, Watanabe K, Noda Y, Ueda K, Asada-Utsugi M, Kubota M, Okawa K, Ihara M, Shimohama S, Uemura K, Kinoshita A (2015) High fat diet enhances β-site cleavage of amyloid precursor protein (APP) via promoting β-site APP cleaving enzyme 1/adaptor protein 2/clathrin complex formation. *PLoS One* **10**, 1–16.

[29] Baig S, Joseph SA, Tayler H, Abraham R, Owen MJ, Williams J, Kehoe PG, Love S (2010) Distribution and expression of picalm in Alzheimer disease. *J Neuropathol Exp Neurol* **69**, 1071–1077.

[30] Tian Y, Chang JC, Fan EY, Flajolet M, Greengard P (2013) Adaptor complex AP2/PICALM, through interaction with LC3, targets Alzheimer’s APP-CTF for terminal degradation via autophagy. *Proc Natl Acad Sci U S A* **110**, 17071–17076.

[31] Zaidi A (2010) Plasma membrane Ca-ATPases: Targets of oxidative stress in brain aging and neurodegeneration. *World J Biol Chem* **1**, 271–280.

[32] Berrocal M, Sepulveda MR, Vazquez-Hernandez M, Mata AM (2012) Calmodulin antagonizes amyloid-β peptides-mediated inhibition of brain plasma membrane Ca(2+)-ATPase. *Biochim Biophys Acta* **1822**, 961–969.

[33] Mata AM, Berrocal M, Sepúlveda MR (2011) Impairment of the activity of the plasma membrane Ca^2+^-ATPase in Alzheimer’s disease. *Biochem Soc Trans* **39**, 819–822.

[34] Green KN, LaFerla FM (2008) Linking calcium to Abeta and Alzheimer’s disease. *Neuron* **59**, 190–194.

[35] Kaja S, Duncan RS, Longoria S, Hilgenberg JD, Payne AJ, Desai NM, Parikh RA, Burroughs SL, Gregg EV, Goad DL, Koulen P (2011) Novel mechanism of increased Ca2+ release following oxidative stress in neuronal cells involves type 2 inositol-1,4,5-trisphosphate receptors. *Neuroscience* **175**, 281–291.

[36] Jiang P, Chen C, Liu X-B, Selvaraj V, Liu W, Feldman DH, Liu Y, Pleasure DE, Li RA, Deng W (2013) Generation and characterization of spiking and non-spiking oligodendroglial progenitor cells from embryonic stem cells Peng. *Stem Cells* **31**, 2620–2631.
