# Supplementary figures and images for: Analytical Strategy to Prioritize Alzheimer’s Disease Candidate Genes in Gene Regulatory Networks Using Public Expression Data
Source: J Alzheimers Dis. 2017 Aug 14;59(4):1237–54. doi: 10.3233/JAD-170011 (PMC5611835; doi:10.3233/JAD-170011)

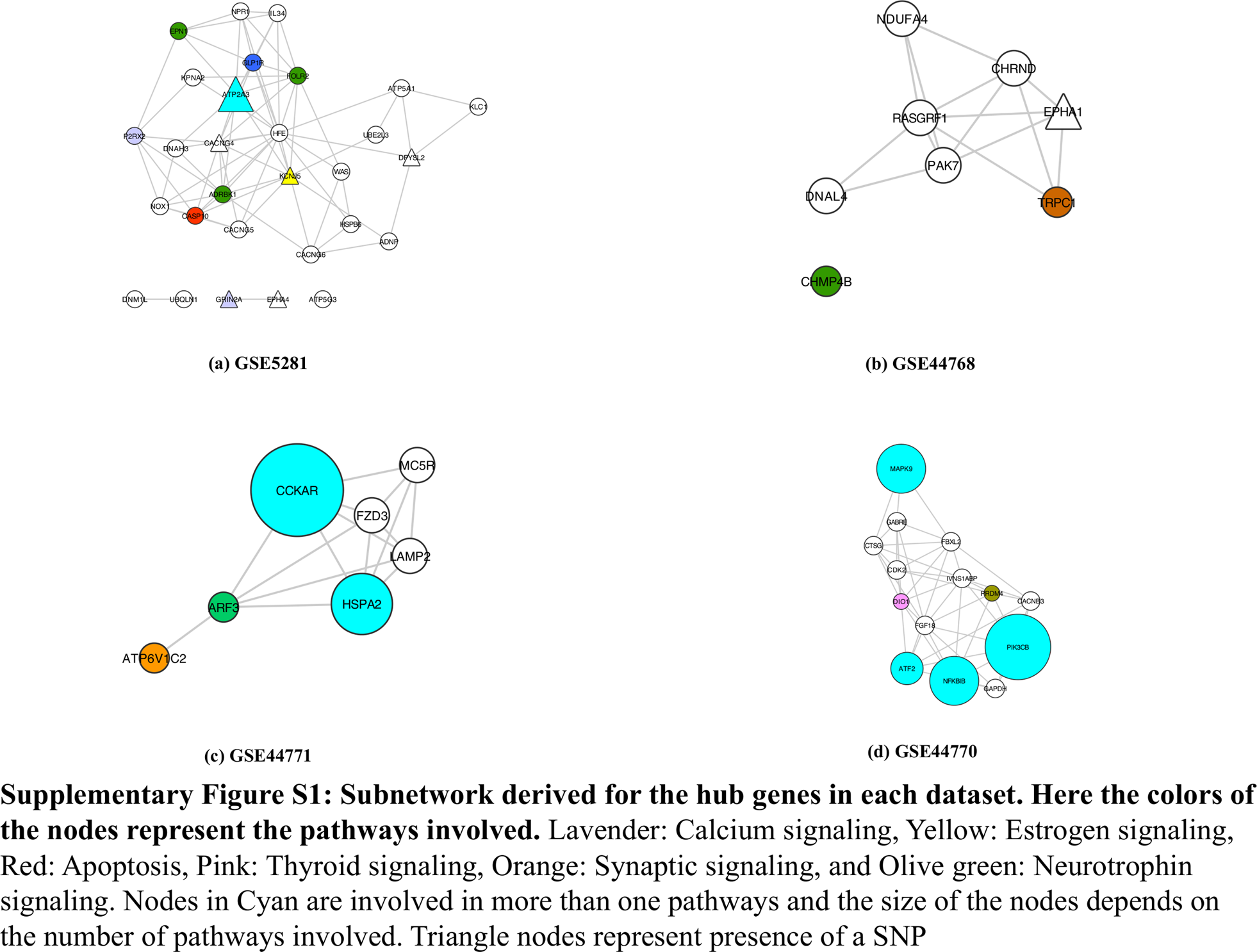

Supplement: Supplementary Figure 1 [file jad-59-jad170011-s007.tif]
